# Propionate‐producing engineered probiotics ameliorated murine ulcerative colitis by restoring anti‐inflammatory macrophage via the GPR43/HDAC1/IL‐10 axis

**DOI:** 10.1002/btm2.10682

**Published:** 2024-05-27

**Authors:** Guangbo Kang, Xiaoli Wang, Mengxue Gao, Lina Wang, Zelin Feng, Shuxian Meng, Jiahao Wu, Zhixin Zhu, Xinran Gao, Xiaocang Cao, He Huang

**Affiliations:** ^1^ Frontiers Science Center for Synthetic Biology and Key Laboratory of Systems Bioengineering (Ministry of Education), School of Chemical Engineering and Technology Tianjin University Tianjin China; ^2^ Frontiers Research Institute for Synthetic Biology Tianjin University Tianjin China; ^3^ Department of Hepato‐Gastroenterology, Tianjin Medical University General Hospital Tianjin Medical University Tianjin China

**Keywords:** anti‐inflammatory macrophages, engineered probiotics, *Escherichia coli* Nissle 1917, inflammatory bowel disease, propionate synthetic biology

## Abstract

Inflammatory bowel disease (IBD) is a chronic and unspecific inflammatory disorder of the gastrointestinal tract, and current treatment options often fail to maintain long‐term remission. Studies have shown that propionate level is reduced in fecal samples from patients with IBD. Propionate can ameliorate IBD through intestinal epithelial cells and immune regulation, but its effects on the inflammatory microenvironment and macrophage differentiation have not been widely studied. To address this, we constructed an engineered propionate‐producing probiotic (EcNP3) to achieve sustained restoration of propionate levels in the gut and increase its bioavailability. DSS‐induced experimental intestinal inflammation model was used to evaluate the effect of EcNP3 on improving the intestinal mucosal barrier and increasing the proportion of anti‐inflammatory macrophages. It was found that EcNP3 exhibited a restorative effect on the depletion of peritoneal anti‐inflammatory macrophages (F4/80hiCD11bhi) and significantly improved the expression level of IL‐10. Simultaneously, the expression of IL‐1β, IL‐6, and CXCL1 was downregulated while inhibiting apoptosis of tissue‐resident macrophages ex vivo. Further investigation revealed that EcNP3 regulates IL‐10 expression through G protein‐coupled receptor 43 and histone deacetylase. Furthermore, EcNP3 significantly inhibited the protein expression of HDAC1 and promoted the histone acetylation level of cells. Finally, EcNP3 significantly improved DSS‐induced colitis in mice by increasing mucus production and reducing inflammatory infiltration. Our results suggest that the engineered live biotherapeutic product EcNP3 is a safe and potently efficacious treatment for IBD, which defines a novel strategy in IBD therapy through macrophage IL‐10 signaling.

**Abbreviations**
3‐HB: 3‐hydroxybutyric acid
CFU: colony forming unit
DAI: disease activity index
DMEM: dulbecco's modified eagle medium
DSS: dextran sulfate sodium
EcN: *E. coli* Nissle 1917
FBS: fetal bovine serum
GPR43: G protein‐coupled receptor 43
H&E: hematoxylin and eosin
HDAC: histone deacetylase.
IBD: Inflammatory bowel disease
IHC: immunohistochemistry
IPTG: isopropyl‐β‐d‐thiogalactoside
LBPs: live biotherapeutic products
LPS: Lipopolysaccharide
PBS: phosphate buffer solution
PMSF: phenylmethylsulfonyl fluoride
PVDF: polyvinylidene fluoride
RIPA: radio immunoprecipitation assay lysis buffer
SCFAs: short‐chain fatty acids
SPF: specific pathogen‐free
TBST: tris buffered saline tween‐20
Tregs: regulatory T cells

**Translational Impact Statement**

Studies have shown that propionate level is reduced in fecal samples from patients with inflammatory bowel disease (IBD). Propionate can ameliorate IBD through intestinal epithelial cells and immune regulation. In this study, we constructed an engineered propionate‐producing probiotic (EcNP3) to achieve sustained restoration of propionate levels in the gut and increase its bioavailability. EcNP3 exhibited a restorative effect on the depletion of peritoneal anti‐inflammatory macrophages (F4/80hiCD11bhi), significantly increased the expression level of IL‐10 in macrophages, and alleviated experimental colitis.

## 1 INTRODUCTION

Inflammatory bowel disease (IBD), a group of intestinal disorders with chronic inflammation, has gradually become a global health burden. Although several drugs appear to relieve IBD‐associated symptoms, long‐term remission cannot be maintained, and clinicians and patients urgently need new therapeutic strategies.^1^ IBD is closely associated with immune dysfunction characterized by excessive and persistent inflammation due to suppressed protective mechanisms in the immune response.^2^ While most research on IBD focuses on abnormal adaptive immunity, recent studies have highlighted the importance of innate immune responses. Macrophages have been identified as a critical player in maintaining the homeostasis of the immune system.^3^, ^4^ Dysregulation of gut macrophage has been demonstrated as a chronic inflammatory trigger for IBD, leading to the loss of intestinal tolerance to commensal bacteria and food antigens.^5^

Macrophages serve as the first line of defense against infectious agents and help maintain mucosal immune system homeostasis by recognizing pathogens, engulfing microorganisms, and regulating intestinal inflammation.^6^ Tissue anti‐inflammatory macrophages play a vital role in maintaining local intestinal homeostasis by eliminating apoptotic or senescent cells and facilitating tissue remodeling.^7^, ^8^ Our previous studies have observed that elevated levels of reactive oxygen species in the colitis region lead to F4/80hiCD11bhi tissue‐resident macrophage depletion, thus contributing to disease progression.^9^ Fortunately, macrophages possess remarkable plasticity, allowing them to alter their phenotype and function based on environmental signals.^10^ Correcting the proportion of macrophage subtypes by targeting macrophage polarization exert a pivotal effect in the resolution of intestinal inflammation and IBD treatment.^11^ Anti‐inflammatory macrophages induce the proliferation of regulatory T cells (Tregs) by secreting the anti‐inflammatory cytokine IL‐10.^12^ Foxp3^+^ Tregs suppress the overactivation of effector T cells, thereby improving mucosal immune tolerance, restoring the intestinal barrier, and maintaining intestinal homeostasis. Moreover, mice with insufficient anti‐inflammatory macrophage exhibit lower tolerance to dextran sulfate sodium (DSS)‐induced colitis.^13^ Hence, these findings suggest that anti‐inflammatory macrophages play a crucial role in colonic inflammation treatment.

Furthermore, the macrophage phenotype appears to be impacted by the drugs used in IBD treatment. For instance, significant M2‐type macrophage induction was observed in patients with mucosal healing after treatment with infliximab, while not present in patients without healing.^14^ Tofacitinib downregulated M1‐type macrophage proportion and promoted M2 polarization with upregulating CD206, CD163, and IL‐10.^15^ Patients with clinical responses to tofacitinib had an increased subset of CD206‐expressing macrophages in the gut compared with non‐responders.^15^ Vedolizumab treatment has also been associated with innate immunity, including changes in macrophage populations and apparent alterations in the expression of molecules involved in macrophage immunity function.^16^ New advances strongly suggest that a defect in the transition of monocytes to mature anti‐inflammatory macrophages in patients with IBD experiences incomplete remission or relapse.^4^ Modulation of macrophage phenotype and associate functions through inhibiting inflammatory signaling pathways and inducing macrophage re‐polarization emerges as a promising approach for IBD treatment.

Short‐chain fatty acids (SCFAs) are essential in regulating mucosal homeostasis and energy metabolism,^17^ among which propionate and butyrate can be directly used as energy sources for colon epithelial cells.^18^ Extensive research has investigated the effects of SCFAs, especially butyrate, on intestinal immunity. SCFAs can regulate the production of inflammatory cytokines, thereby mediating the host immune responses. Direct exposure of mouse macrophages to butyrate has been shown to downregulate the production of LPS‐induced proinflammatory cytokines by inhibiting histone deacetylase.^19^ Butyrate has been proven to promote the alternative activation of intestinal macrophages to restore intestinal immune homeostasis. Moreover, the depletion of SCFAs during antibiotic administration has resulted in the hyperresponsiveness of murine intestinal macrophages to bacterial stimuli, leading to T‐cell dysfunction.^20^ Propionate is one of the most abundant SCFAs, which can regulate various immune cells. It can regulate lymphocyte proliferation and metabolism, inhibit antigen‐specific CD8^+^ T cell activation, and induce apoptosis and degranulation of human basophils.^21^, ^22^, ^23^, ^24^ However, there is currently limited information on the effects of propionate on anti‐inflammatory macrophages and their functions.

Propionate administrated orally is quickly metabolized and not efficiently delivered to the colon. It may also affect the metabolism and homeostasis of the body, posing potential risks to human health. Currently, some emerging methods address this problem, such as encapsulating propionate using biomaterials or using live biotherapeutic products (LBPs) to achieve the ideal concentration of propionate in the colon. However, the release rate and degradation rate of propionate in the intestine can be influenced by the properties of biomaterials, intestinal pH, and intestinal microbiota, thereby affecting its bioavailability and efficacy. Engineered LBPs (eLBPs) are living organisms used to treat, cure, or prevent human diseases or conditions.^25^ They can accurately and controllably accomplish in vivo diagnosis and treatment with fewer side effects. In recent years, numerous studies have confirmed the therapeutic effects of eLBPs on colitis,^26^, ^27^ phenylketonuria,^28^ hyperammonemia,^29^ obesity,^30^, ^31^ depression,^32^ and cancer.^33^ Many clinical trials of eLBPs are ongoing, indicating their broad application prospects as a new microbial therapy.

**SCHEME 1:**
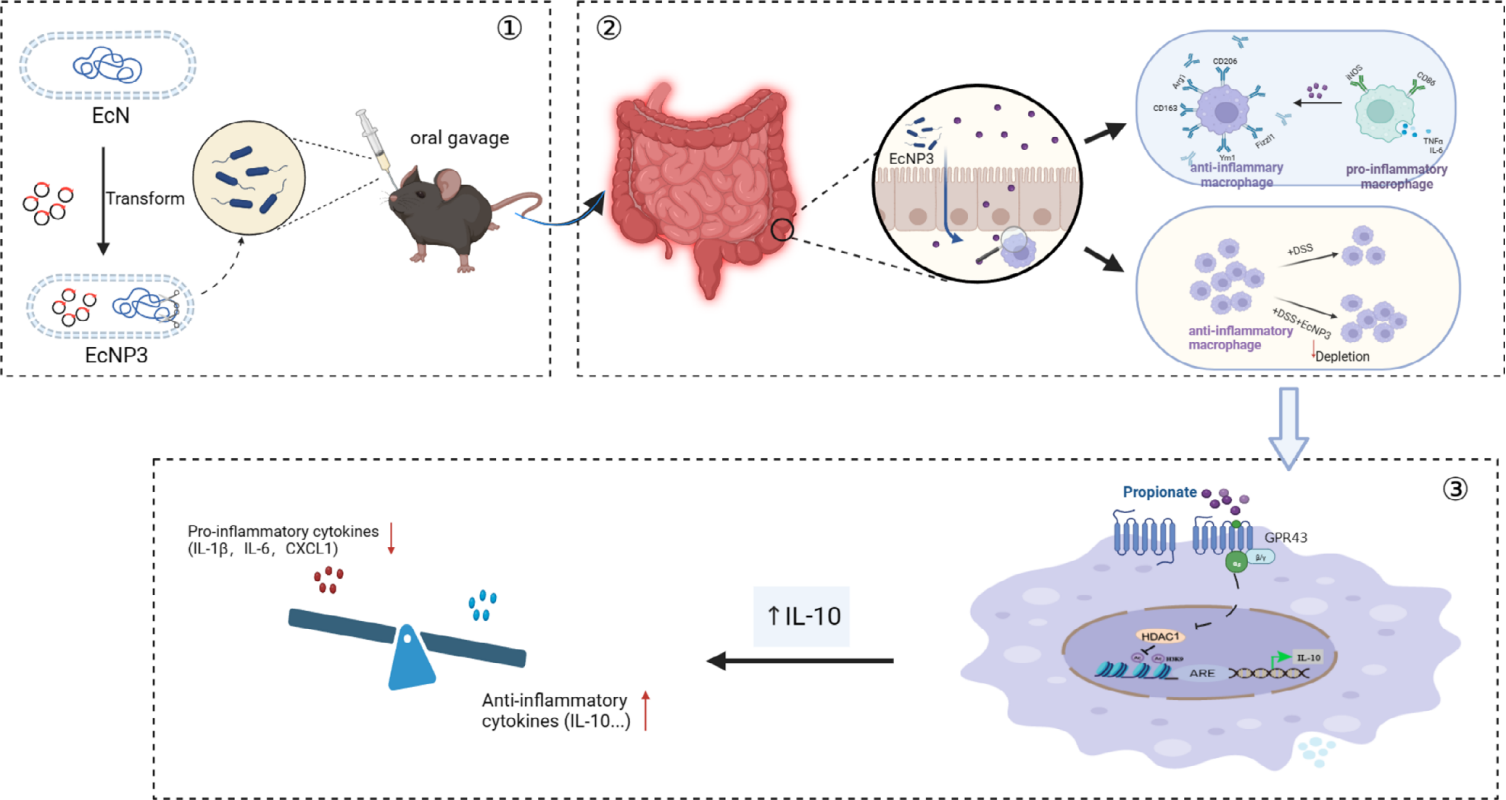
Schematic illustration of the EcNP3 therapeutic.

In this study, we investigated the impact of propionate on macrophage phenotype and used synthetic biological approaches to construct propionate‐producing engineered probiotics to further explore its effect on anti‐inflammatory macrophages and its potential role in IBD treatment (Scheme 1).

## 2 MATERIALS AND METHODS

### 2.1 Strains and culture conditions

*Escherichia coli* Nissle 1917 (EcN) was purchased from the National standard material resource platform (www.biobw.com) (Biobw, China). *E. coli* DH5a (TransGen, China) was used for DNA cloning and plasmid amplification. EcN and *E. coli* DH5a were cultured in Luria‐Bertani broth or on agar plates at 37°C. The concentrations of kanamycin, spectinomycin, ampicillin, and streptomycin were 50, 50, 100, and 50 μg/mL.

### 2.2 Engineered propionate‐producing bacteria construction

Plasmids used in this study are listed in Table S1. Oligonucleotides used as primers are listed in Table S2. Genes *IdhA*, *pct*, *lcdA*, and *acul* were synthesized and optimized by Genewiz (China). The lactate synthase gene *ldhA* is derived from EcN. The propionate coenzyme A‐transferase gene *pct* is derived from *Moorella thermoacetica*. The lactyl CoA dehydratase gene *lcdA* and the acyl CoA reductase gene *acuI* are derived from *Clostridium propionate*. The above genes were inserted into vectors pCDFDuet and pETDuet, respectively. pETDuet and pCDFDuet‐1 plasmids are IPTG inducible promoters. The IPTG inducible promoters were replaced with the constitutive promoters J23119. Then, the vectors containing foreign genes were introduced into EcN to construct EcNP1.

The genes related to acetic acid synthesis and metabolism were knocked out by using the CRISPR‐Cas9 double plasmid system.^34^ First, according to the target gene, the N20 sequence (Table S2) was designed using the online software Cas‐OFFinder (http://www.rgenome.net/cas-offinder/). Select the homologous region of 250–500 bp before and after the target gene to construct the homologous arm. The L fragment was amplified with pTargetF‐L primer and L‐sgRNA primer using the original pTargetF as the template. Then, the S fragment was amplified with pTargetF‐R primer and corresponding primer S‐sgRNA. Then, the L segment, homologous arm, and the S segment were connected to construct the pTargetF plasmid targeting specific genes. The plasmid with the correct sequence was selected and introduced into the strain containing pCas plasmid for gene knockout.

### 2.3 Propionate analysis

Gas chromatography–mass spectrometry is used to detect propionate in the fermentation broth. Centrifuge the fermentation broth at 12 000 rpm for 2 min. Then add 1 mL of 50% H_2_SO_4_ and 2–3 mL of ether to 5 mL of supernatant and shake for 45 min. Add an appropriate amount of anhydrous calcium chloride to the acidified fermentation broth to remove the water. The remaining liquid passes through a 0.22 μM filtration membrane filtration. Agilent gas chromatography‐triple quadrupole mass spectrometer was used for detection. The injection volume is 1 μL. The split ratio is 20:1. The cleaning solution is dichloromethane and ether. Clean the injection probe twice with ether before injection and twice with dichloromethane after injection. The carrier gas flow is 1 mL/min. The temperature of the electron collision source is 230°C with an electron energy of 70 eV. The quadrupole temperature is 150°C, and the interface temperature between GC and MS is 250°C. MS temperature process: initial 60°C, hold for 2 min, rise to 220°C at the rate of 10°C/min, and hold for 20 min.

### 2.4 In vitro fermentation model

Simulate the colonic fermentation environment by using colonic fluid with different pH values. The simulated intestinal fluids were made according to the previously published procedures.^35^ Simulate the proximal and distal colon by adjusting the pH value (pH 5.6 and pH 6.3). Inoculate engineered strains into the fermentation broth and supernatants are taken every 8 hours to detect the concentration of PA.

### 2.5 Cell culture

RAW264.7 (Procell, China) was cultured in DMEM (Gibco, Thermo Fisher Scientific, USA) complete medium containing 10% fetal bovine serum (FBS). Cells were incubated in an incubator containing 5% CO_2_ at 37°C. The cells were passaged 1:2 every 1–2 days.

### 2.6 Animal experiments

In total, 6–8 weeks C57BL6/J male mice (Vital River, China) were housed in an SPF condition under 12 h light /12 h dark and fed ad libitum, and experimental intervention was performed after 1 week of adaptive feeding. All animal welfare and experimental procedures were in accordance with the Regulations on the Administration of Laboratory Animals in China and the relevant ethical regulations of the Tianjin Medical University.

In the study on the survival of EcNP3 in the gastrointestinal tract of healthy mice, the C57BL/6J mice were randomly divided into groups EcN (containing empty plasmids pETDuet and pCDFDuet‐1 plasmids) and EcNP3. The mice were orally administered EcN (5 × 10^7^ CFU) or EcNP3 (5 × 10^7^ CFU) on the first day. The content of EcN and EcNP3 in feces was determined daily with a plate containing ampicillin selectively. In the study on EcNP3 alleviated the symptoms of DSS‐induced colitis, C57BL6/J mice were randomly divided into 5 groups: Control group, DSS group, DSS + EcNP3 group, DSS + EcN group, and DSS+ Propionate group. Mice were pretreated with EcNP3, EcN, and sodium propionate for 3 days. The other four groups were given 3% DSS (MP Biomedicals, USA) in drinking water for 7 days, except for the control group. At the same time, the DSS + EcNP3 group, DSS + EcN group and DSS + propionate group were intervened with EcNP3 (5 × 10^7^ CFU), EcN (5 × 10^7^ CFU) and sodium propionate (200 mM), respectively, and the control and DSS groups were intervened with PBS. The body weight, fecal occult blood, and fecal characteristics of mice were monitored and recorded daily. The disease activity index (DAI) of mice was scored according to Table S3. On Day 11, mice were sacrificed by cervical dislocation under anesthesia, blood was collected, the colon was removed, and length was measured. Tissues were fixed in 4% paraformaldehyde solution or stored at −80°C for further analysis.

### 2.7 Macrophage isolation and treatment

After the mice were sacrificed by cervical dislocation under anesthesia, 5 mL serum‐free DMEM medium was injected into the abdominal cavity. The abdomen of the mice was gently rubbed for 3 min and left for 15 min. The intra‐abdominal fluid was aspirated into a centrifuge tube with a sterile syringe. The cells were centrifuged at 450*g* for 5 min and washed twice with PBS. The mixed red blood cells in the cells were lysed with red cell lysis buffer to obtain leukocyte precipitation. The obtained cells were filtered using a 0.7 μm cell filter. Then, the cells were analyzed by flow cytometry directly or seeded in a complete DMEM medium containing 10% FBS and 1% Streptomycin penicillin in the incubator. After overnight culture, nonadherent cells were removed and washed twice with PBS. The corresponding experimental intervention was given for 24 h, and flow cytometry analysis or RNA extraction was performed.

### 2.8 Flow cytometry analysis

The cells obtained from the peritoneal cavity or cultured cells were prepared into single‐cell suspension. The number of cells in each flow tube was adjusted to 10^6^ cells/tube. After centrifugation at 300*g* for 5 min, the supernatant was discarded, washed once with cell staining buffer, and resuspended cells in 100 μL buffer. A total of 2 μL TruStain FcX™ reagent (Biolegend, San Diego, USA) was added to each tube and incubated for 15 min on ice. Then, 1 μL Zombie UV reagent was added and incubated in the dark for 15 min at room temperature. The surface antibodies APC/Cy7, anti‐mouse F4/80 antibody, and PE‐Texas Red anti‐mouse CD11b antibody (Biolegend, San Diego, USA) were further incubated at 4°C for 30 min in the dark. Cells were washed with cell staining buffer, centrifuged for 5 min, and the supernatant was discarded. Cell staining buffer was added to resuspend the cells and quantified.

### 2.9 H&E staining and immunohistochemistry analysis

The colon tissues fixed in 4% paraformaldehyde solution were paraffinized and sectioned (5 mm thick). Then, the sections were stained with hematoxylin and eosin (H&E). For immunohistochemistry, the sections were deparaffinized and rehydrated, processed with microwave antigen retrieval, and incubated overnight with primary antibodies against MUC2 (ab272692) (Abcam, Cambridge, UK), ZO‐1 (21773‐1‐AP), Occludin (27260‐1‐AP) (Proteintech, China), Claudin 2 (CSB‐PA070222), and Claudin5 (CSB‐PA001672) (Cusabio, China) at 4°C. They were further incubated with secondary antibodies and streptavidin–horseradish peroxidase with diaminobenzidine.

### 2.10 Cytokine analysis

Multiple inflammatory cytokines were quantified in single samples using a 10‐Plex Mouse magnetic capture bead cytokine panel (Invitrogen, Thermo, USA), read in a FlexMap 3D instrument, and analyzed using xPONENT 4.2 (Luminex, USA).

### 2.11 Real‐time PCR

Total RNA was extracted from mouse colon or cells using Trizol reagent, and the obtained RNA was reverse transcribed into cDNA using HiScript Q RT SuperMix (Vazyme, China), as per the manufacturer's instructions. For quantitative comparisons, cDNA samples were analyzed by real‐time PCR using the ChamQ SYBR Color qPCR Master Mix (Vazyme, China) on the QuantStudioTM 3 real‐time PCR instrument (Thermofisher, USA). The QuantStudio™ design and analysis SE software is used to process data according to the instructions. After the cDNA underwent three stages of pre‐denaturation, cycling reaction, and dissolution curve, the CT value was analyzed. And 2^−△△CT^ was calculated, which represents the expression of mRNA. Primer sequences are presented in Table S4.

### 2.12 Western blot

Mouse colon or cells were lysed in protein lysis mix (RIPA: PMSF: protein phosphatase inhibitor = 100: 1: 1) for 30 min, centrifuged at 12000 rpm for 15 min at 4°C, and the supernatant was retained. Protein concentration was determined using the BCA kit. The protein loading buffer was added to the protein liquid, and the protein was denatured at 100°C for 10 min. A total of 20 μg protein was added to SDS‐PAGE gel for separation, and the protein was transferred to a 0.22 mm PVDF membrane by wet transfer at 90 V for 90 min. Then, PVDF membranes were blocked in TBST buffer containing 5% skim milk for 90 min at room temperature, followed by incubation with primary antibodies, anti‐HDAC1 antibody (ab109411), anti‐Histone H3 antibody (ab1791), anti‐Histone H3(acetyl K14) (ab52946) (Abcam, Cambridge, UK), iNOS Recombinant antibody (80517‐1‐RR), Arginase‐1 Monoclonal antibody (66129‐1‐Ig), CD163 Polyclonal antibody (16646‐1‐AP), and anti‐β‐actin recombinant antibody (66009‐1‐Ig) (Proteintech, China), overnight at 4°C. After washing three times with TBST, the membranes were incubated with goat anti‐mouse IgG (SA00001‐1) or goat anti‐rabbit IgG (SA00001‐2) (Proteintech, China) secondary antibodies for 60 min at room temperature. Finally, proteins were detected by chemiluminescence. The grayscale of the proteins was quantified and analyzed using ImageJ software.

### 2.13 Enzyme‐linked immunosorbent assay

Enzyme‐linked immunosorbent assay (ELISA) is used to detect the expression levels of inflammatory factors in the culture medium of the treated cells. Mouse mononuclear macrophage RAW264.7 were treated with LPS (100 ng/mL) or LPS (100 ng/mL) & Propionate (20 mM). Mouse interleukin‐10 ELISA kit (E‐EL‐M0046c) and Mouse Tumor Necrosis Factor‐α ELISA Kit (E‐EL‐M3063) (Elabscience, China) were used to detect the cytokine levels in cell culture medium. In addition, RAW264.7 were treated with Propionate (20 mM), Recombinant Mouse IL‐4 Protein (20 ng/mL) (abs04697, absin, China) and Propionate & IL‐4 to investigate the effect of propionate on the anti‐inflammatory macrophages. Mouse interleukin‐10 ELISA kit was used to detect the cytokine levels in the cell culture medium, and the absorbance was measured at OD_450_ using a Varioskan LUX microplate reader (Thermo Scientific).

### 2.14 Statistical analysis

GraphPad Prism 8.3.0 software was used for statistical analysis. All data are expressed as mean ± SD. An independent sample t‐test was used to compare the two groups, and one‐way ANOVA was used to analyze the differences between groups. *p* < 0.05 was considered statistically significant.

## 3 RESULTS

### 3.1 Propionate promoted the anti‐inflammatory macrophage phenotype

**FIGURE 1:**
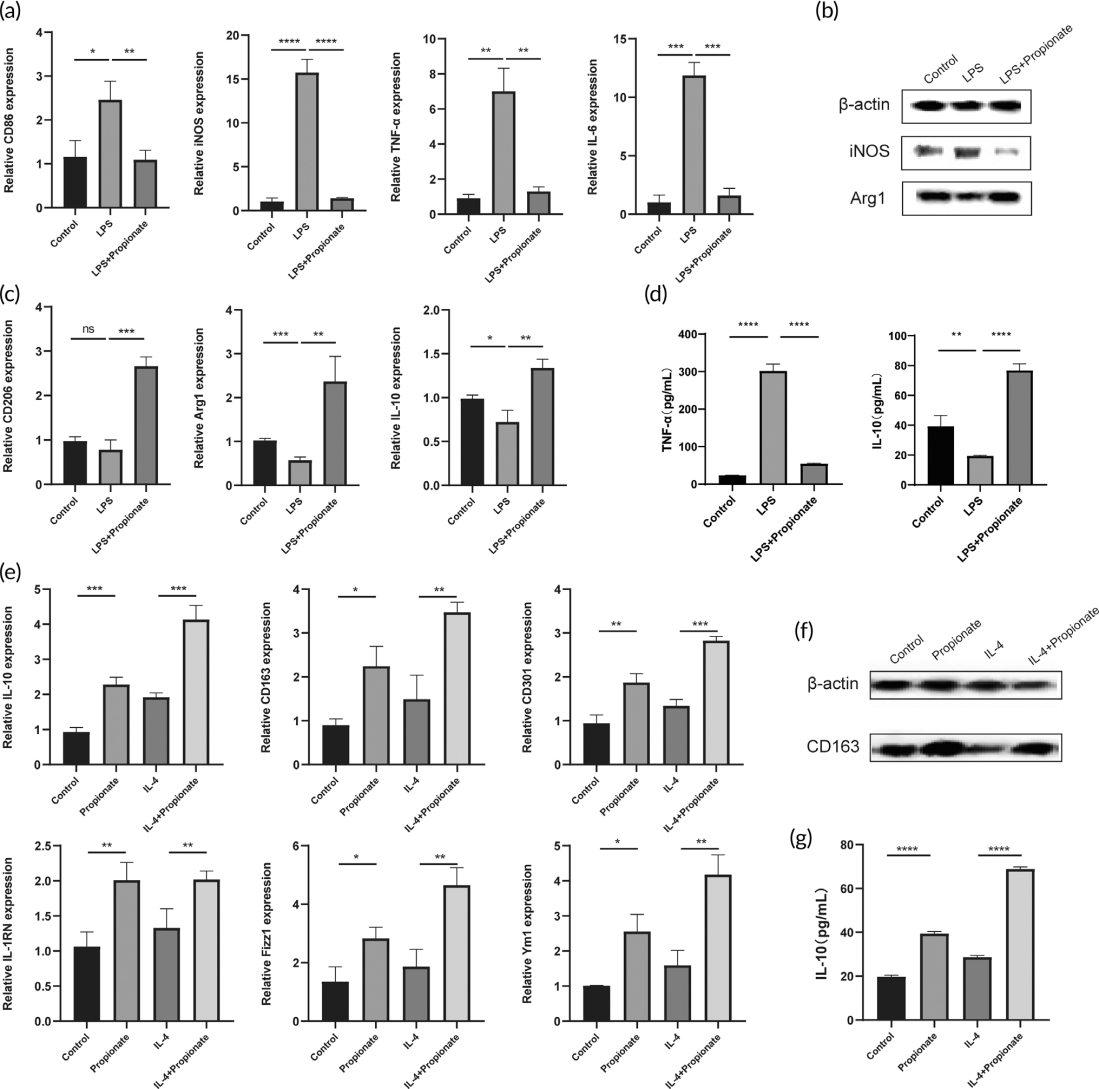
Propionate promoted anti‐inflammatory macrophage phenotype and inhibited pro‐inflammatory macrophage phenotype. (a) Propionate downregulated pro‐inflammatory macrophage‐related factors under LPS conditions. (b) Western blot analysis of the iNOS and Arg1 expression levels under LPS or LPS + Propionate conditions. (c) Propionate upregulated anti‐inflammatory macrophage‐related factors under LPS conditions. (d) Enzyme‐linked immunosorbent assay analysis of the TNF‐α and IL‐10 expression levels under LPS or LPS + Propionate conditions. (e) Propionate promoted the anti‐inflammatory macrophage phenotype by promoting M0 toward the anti‐inflammatory type and enhanced the expression of the anti‐inflammatory type under IL‐4 conditions. (f) Propionate promoted the expression of CD163. (g) Propionate promoted the expression level of IL‐10 in macrophages, especially under IL‐4 conditions. Statistical analysis between the two groups was performed via an independent sample *t*‐test. *p < 0.05; **p < 0.01; ***p < 0.001; ****p < 0.0001, ns, no significance.

Macrophages show notable plasticity, which responds to stimuli in the environment and changes into different phenotypes.^10^ We investigated the effect of propionate on the phenotype of RAW 264.7. We used lipopolysaccharide (LPS) (Thermo Fisher Scientific, USA) to induce the formation of pro‐inflammatory macrophages and found that propionate could reduce the expression of the inflammatory markers CD86 and iNOS, as well as the expression of the pro‐inflammatory cytokines TNF‐α and IL‐6 in RAW264.7 (Figure 1a,b,d). At the same time, anti‐inflammatory subtype markers were detected under LPS stimuli, and the results showed that propionate upregulated the expression of CD206, Arg1, and IL‐10 in RAW264.7 (Figure 1b–d). Moreover, we explored the effect of propionate on anti‐inflammatory macrophages and found that propionate not only promoted the development of primitive macrophages (M0) to an anti‐inflammatory phenotype but also enhanced the expression of anti‐inflammatory macrophage markers in IL‐4‐induced RAW264.7. In addition, the expression of anti‐inflammatory markers IL‐10, CD163, IL‐1RN, Fizz1, Ym1, and CD301 showed a consistent trend (Figure 1e–g). These results suggested that propionate can promote the development of macrophages toward an anti‐inflammatory phenotype.

### 3.2 Construction of engineered propionate‐producing probiotics

**FIGURE 2:**
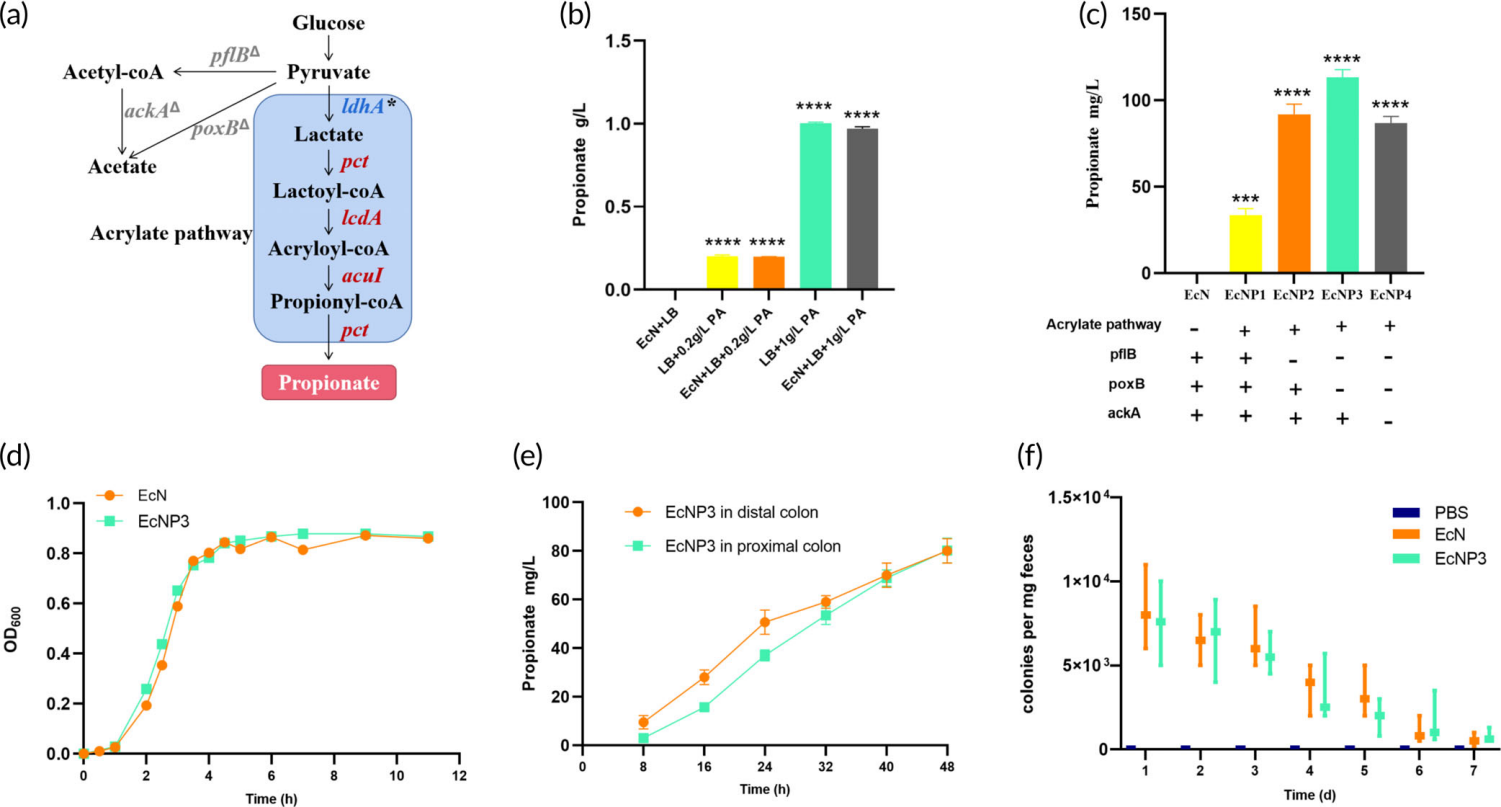
Construction and characterization of engineered EcN. (a) Metabolic pathway of propionate. *ldhA*, the key gene of lactic acid synthesis, was overexpressed. Genes pct, *lcdA*, and *acuI* were introduced. Genes *pflB*, *poxB*, and *ackA* were knocked out. (b) The effect of EcN on propionate metabolism. (c) The amount of propionate produced by EcN, EcNP1, EcNP2, EcNP3, and EcNP4. (d) The growth curve of EcN and EcNP3. (e) The amount of propionate produced by EcNP3 in vitro simulated colon fermentation model. The pH value is used to distinguish the distal end from the proximal end. The simulated pH of the proximal end is 5.6 and that of the distal end is 6.3. (f) Gut colonization dynamics of engineered EcN after oral administration. Mice were treated with EcN and EcNP3 on Day 1. Fecal EcN and EcNP3 were determined daily using selective ampicillin‐containing plates. Statistical analysis was performed via one‐way ANOVA. *p < 0.05; **p < 0.01; ***p < 0.001; ****p < 0.0001.

Given the low bioavailability and duration of propionate for direct use,^36^ we constructed engineered probiotics to increase propionate production and exert a long‐lasting role in the gastrointestinal tract. EcN was selected as a chassis for propionate production due to its compatibility with effective synthetic biology tools and long track record of safety.^37^ First, we found that EcN lacked the key genes for propionate synthesis (Figure 2a) and could not degrade propionate as a rich potential source of carbon (Figure 2b). Therefore, we constructed the engineered strain EcNP1 by introducing the biosynthesis pathway of propionate in EcN (Figure 2a). EcN was transformed with plasmids pETDuet‐*IdhA*‐*pct* and pCDFDuet‐*lcdA*‐*acul* containing the genes encoding lactate dehydrogenase (LDH), propionate CoA‐transferase (PCT), lactoyl‐CoA dehydratase (LCD), and acryloyl‐CoA reductase (ACU) under the promoter J23119. The cell‐free supernatants of EcNP1 were collected separately and detected by GC–MS. As expected, the recombinant strain EcNP1 generated 33.38 ± 4.38 mg/L propionate (Figure 2c).

Acetate is a key by‐product in the process of propionate synthesis. Knocking out genes related to acetate synthesis and metabolism is one of the strategies to improve the ability to synthesize propionate. We used the CRISPR‐Cas9 double plasmid system to knock out the genes *pflB*, *poxB*, and *ackA* related to acetate synthesis within the EcNP1 genome to construct EcNP2, EcNP3, and EcNP4. Among them, the simultaneous knockout of *pflB* and *poxB* effectively weakened acetate synthesis and increased propionate production (EcNP3, 113.23 ± 5.17 mg/L) (Figure 2c). However, knockout of *ackA* did not significantly improve the synthesis ability of propionate (EcNP4, 86.76 ± 5.01 mg/L). In addition, the introduction and knockout of genes had no significant effect on the growth of probiotics EcN (Figure 2d). Therefore, we selected EcNP3 as the final detection object.

### 3.3 Characterization of engineered propionate‐producing probiotics

Considering that the complex gastrointestinal environment often affects the metabolic pathways of microorganisms, we investigated the ability of EcNP3 to produce propionic acid in the simulated colon environment. EcNP3 was cultured in simulated distal and proximal colon environments, respectively. The content of propionic acid in the supernatant was measured every 8 h. The results showed that the engineered probiotics still have a good synthesis ability of propionate in the simulated fermentation environment within 48 h (Figure 2e) colon environment. Afterward, we also studied the survival of EcNP3 in the gastrointestinal tract of healthy mice. C57BL/6J mice were orally administered EcN or EcNP3 on the first day. The content of EcN and EcNP3 in feces was determined daily with a plate containing ampicillin selectively. EcN and EcNP3 can be retained in the gastrointestinal tract for no more than 7 days (Figure 2f). After administration, the number of intestinal colonies decreased every day. Therefore, the engineered probiotics were administered daily in the follow‐up animal experiments.

### 3.4 EcNP3 alleviated the symptoms of DSS‐induced colitis

**FIGURE 3:**
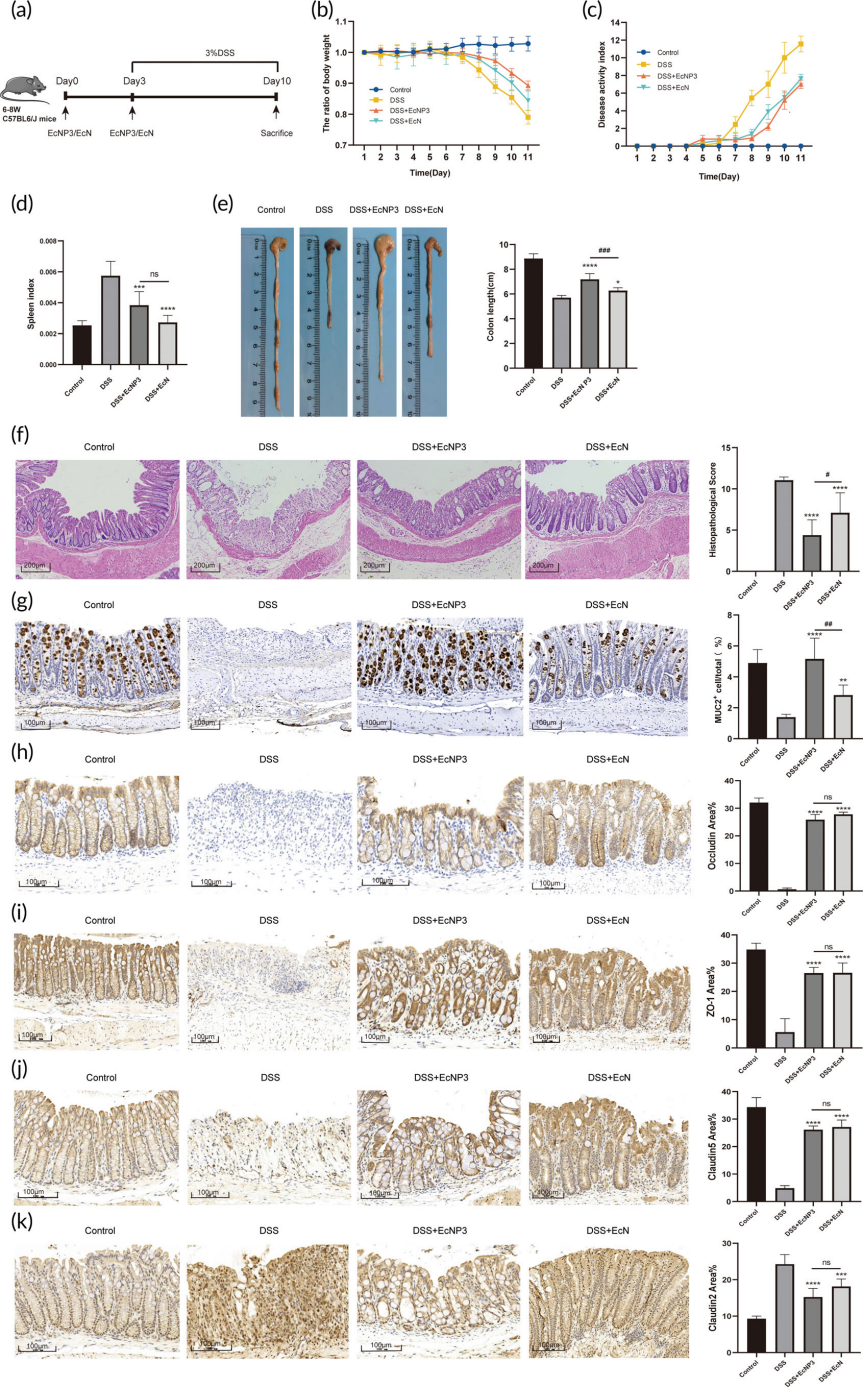
Effects of engineered propionate‐producing bacteria (EcNP3) and EcN on DSS‐induced mouse colitis. (a) Schematic diagram of mouse colitis. (b) The body weight of mice was evaluated in the experiment. (c) The disease activity index was measured daily. (d) The spleen index was calculated. (e) The colon length was measured. (f) The distal colon was stained with hematoxylin and eosin to determine the degree of inflammation. (g–k) The expression of MUC2, Occludin, ZO‐1, Claudin5, and Claudin2 was confirmed using an immunohistochemistry assay. Statistical analysis was performed using one‐way ANOVA compared with the DSS group mice. Error bars represent standard errors. Values represent the mean ± SD of the mean. *p < 0.05; **p < 0.01; ***p < 0.001; ****p < 0.0001. ns, no significance. The DSS + EcNP3 group versus the DSS + EcN group, ^**#**^ *p* < 0.05; ^**##**^ *p* < 0.01; ^**####**^ *p* < 0.0001; ns, no significance.

The DSS‐induced colitis model is a commonly used animal model to mimic the development of IBD (Figure 3a). It is related to immune dysfunction, intestinal flora imbalance, and the toxicity of DSS. DSS‐induced experimental colitis models were used to evaluate the potential of EcNP3 for IBD treatment. EcNP3 significantly alleviated the weight loss and increased disease activity index caused by DSS‐induced colitis (Figure 3b,c). Moreover, the spleen index, which reflects the degree of inflammation, also showed the effectiveness of EcNP3 in attenuating colitis (Figure 3d). The colon shortening was positively correlated with colon inflammation and edema of DSS‐induced colitis. The EcNP3 intervention group reduced the degree of colon shortening in mice and was better than the EcN intervention (Figure 3e). In addition, EcNP3 improved crypt loss and crypt structure disorder in parallel with increasing the number of goblet cells (Figure 3f), these efforts appeared to be better than that of EcN. Mucin‐2 (MUC‐2) is a glycoprotein secreted by goblet cells, which can maintain the structure and function of the mucus and participate in the immune regulation of intestinal mucosa. The immunohistochemistry stain result showed that EcNP3 increased the number of MUC‐2^+^ goblet cells (Figure 3g **)**. In addition, EcNP3 and EcN treatment effectively improved intestinal integrity and tight junction protein expression levels (Figure 3h–k). These results commonly indicated that EcNP3 could relieve colitis symptoms and maintain mucous layer stability.

### 3.5 EcNP3 modulated the immune response in DSS‐induced colitis

**FIGURE 4:**
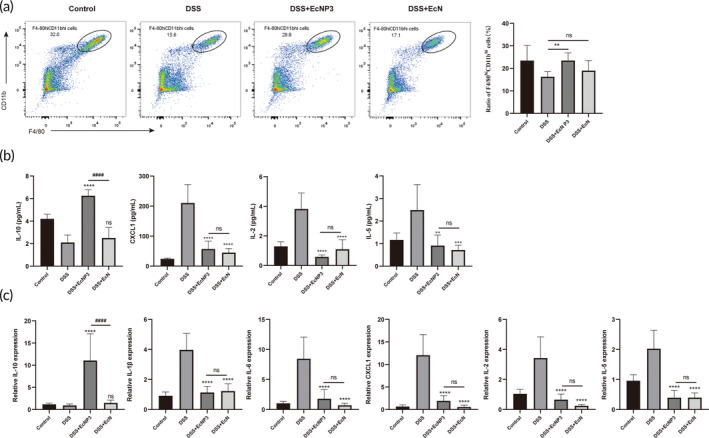
Effects of engineered propionate‐producing bacteria (EcNP3) and *E. coli* Nissle 1917 (EcN) on inflammatory condition. (a) The percentages of F4/80^hi^CD11b^hi^ macrophage after the in vivo experiment were analyzed by flow cytometry. (b) The serum was measured using the Luminex mouse inflammatory cytokine panel. IL‐10, CXCL1, IL‐2, and IL‐5 were measured, while the concentrations of other cytokines in mouse serum were below the detection range. (c) The gene expressions of cytokines in the colon were measured. Statistical analysis was performed using one‐way ANOVA compared with the DSS group mice. Error bars represent standard errors. Values represent the mean ± SD of the mean. *p < 0.05; **p < 0.01; ***p < 0.001; ****p < 0.0001. The DSS + EcNP3 group versus the DSS + EcN group, ^**#**^ *p* < 0.05; ^**##**^ *p* < 0.01; ^**###**^ *p* < 0.001; ns, no significance.

The regulation of EcNP3 on anti‐inflammatory macrophages and cytokines balance in DSS‐induced mice were evaluated. F4/80^hi^CD11b^hi^ macrophages are generally considered to be an anti‐inflammatory phenotype and depletion in inflammatory conditions. Flow cytometry showed that EcNP3 restored the F4/80hiCD11bhi macrophage proportion in peritoneal from mice with colitis (Figure 4a). Classic inflammatory‐related cytokines levels in serum were measured (Figure 4b). EcNP3 significantly increased the concentration of anti‐inflammatory cytokine IL‐10. However, the inhibition of pro‐inflammatory cytokines CXCL1, IL‐2, and IL‐5 was no significant difference between EcNP3 and EcN. Further, corresponding cytokines in the mouse colon tissue were evaluated (Figure 4c), and EcNP3 had significant effects on the expression levels of IL‐10, IL‐1β, IL‐6, CXCL1, IL‐2, and IL‐5. Compared with platform strain, EcNP3 significantly promoted the expression of IL‐10 in serum and intestinal tissue. However, the inhibitory effect of pro‐inflammatory factors was similar. These results indicated that EcNP3 exhibited a critical role in regulating cell immune response in DSS‐induced colitis. Remarkable is the effect of EcNP3 on the significant enhancement of IL‐10 expression level in immune regulation.

### 3.6 EcNP3 modulated macrophages in vitro

**FIGURE 5:**
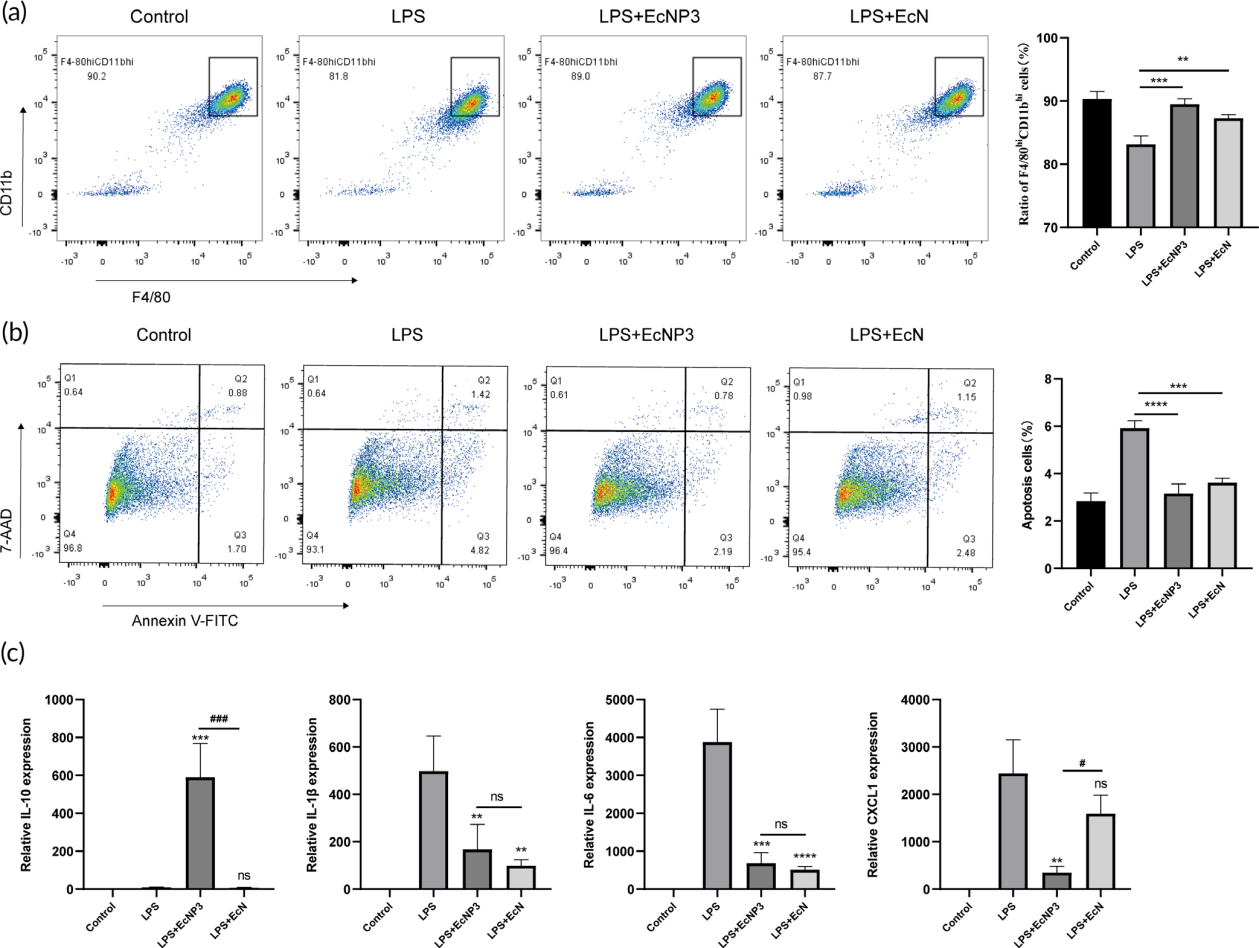
Effects of engineered propionate‐producing bacteria (EcNP3) on the proportion and the immune function of resident macrophage in vivo and in vitro. (a) The ratio of F4/80^hi^CD11b^hi^ cells after in vitro stimulation. (b) The proportions of apoptotic cells were measured. (c) The mRNA expressions of cytokines in cells were measured. Statistical analysis was performed using one‐way ANOVA. Error bars represent standard errors. Values represent the mean ± SD of the mean. *p < 0.05; **p < 0.01; ***p < 0.001; ****p < 0.0001.

Next, we proceeded to investigate the impact of EcNP3 on anti‐inflammatory macrophages through in vitro experiments by peritoneal macrophages from naïve mice. Initially, the effect of EcNP3 on the proportion of peritoneal F4/80^hi^CD11^hi^ macrophages in the context of inflammation was evaluated, and the results were consistent with in vivo experiments (Figure 5a). To further confirm this finding, the impact of EcNP3 on macrophage apoptosis was analyzed. The results indicated that EcNP3 fermentation supernatant could significantly reduce cell apoptosis, including early and late apoptosis (Figure 5b). Additionally, we assessed the influence of EcNP3 on inflammatory cytokines produced by peritoneal macrophages in vitro. The results demonstrated that EcNP3 supernatant significantly upregulated the expression of IL‐10 mRNA and downregulating the expression of IL‐1β, IL‐6, and CXCL1 mRNA (Figure 5c). These findings indicated that EcNP3 could regulate anti‐inflammatory macrophages by influencing their number and cytokine production ability.

### 3.7 The regulatory effect of EcNP3 on IL‐10 depended on G protein‐coupled receptors

**FIGURE 6:**
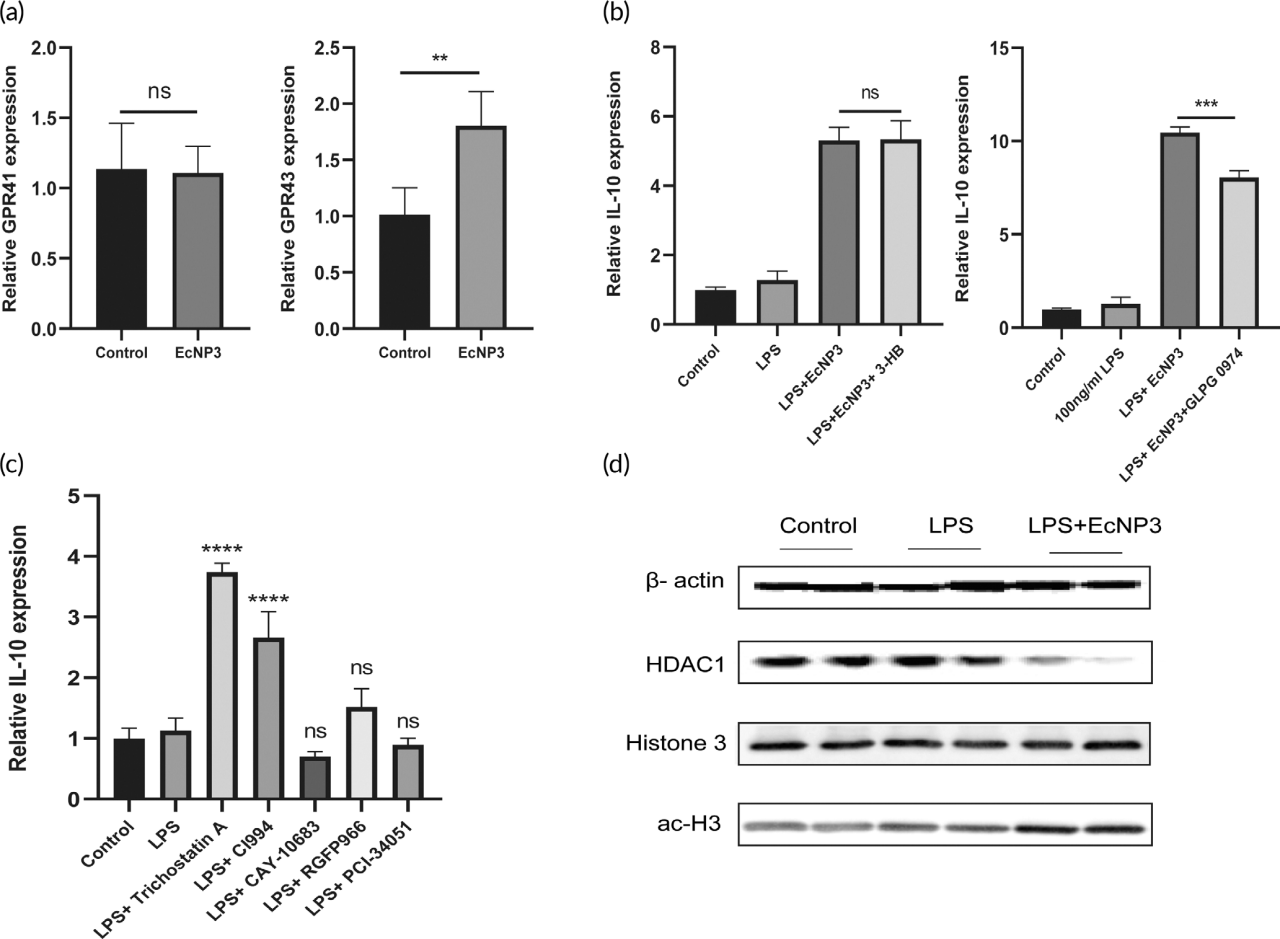
Mechanism of engineered propionate‐producing bacteria (EcNP3) promoted IL‐10 production. (a) EcNP3 supernatant treatment upregulated GPR43 mRNA expression. (b) GPR43 antagonist GLPG 0974 downregulated the effect of EcNP3 on IL‐10. (c) pan‐HDAC inhibitor and HDAC1 inhibitor upregulated IL‐10 mRNA expression. (d) EcNP3 inhibited HDAC1 expression and upregulated the level of histone acetylation. Statistical analysis was performed using one‐way ANOVA, compared with the LPS group. Error bars represent standard errors. Values represent the mean ± SD of the mean. *p < 0.05; **p < 0.01; ***p < 0.001; ****p < 0.0001, ns, no significance.

Interleukin‐10 (IL‐10) is an anti‐inflammatory cytokine that plays an essential role in regulating the immune response and clearing chronic inflammation, and mice with IL‐10 deficiency can develop spontaneous colitis.^38^ The previous experimental results showed that propionate and EcNP3 can significantly upregulate the expression of IL‐10 in anti‐inflammatory macrophages. G protein‐coupled receptor 41 (GPR41, FFA3) or G protein‐coupled receptor 43 (GPR43, FFA2) plays a vital role in intestinal physiology and pathophysiology as ligands of SCFAs.^39^ In this study, mouse macrophage cell line RAW264.7 was used to explore the correlation and role of G‐protein coupled receptors (GPCRs) in EcNP3's promotion of IL‐10 secretion from anti‐inflammatory macrophages. Initially, it was observed that the expression of GPR43 was significantly upregulated by treatment with EcNP3 (Figure 6a). After that, 3‐hydroxybutyric acid (3‐HB, 5 mM) and GLPG 0974 (2 μM) were used to block the GPR41 and GPR43, respectively. The results showed that EcNP3 fermentation supernatant significantly upregulated IL‐10 transcription levels, whereas GLPG0974 effectively blocked this effect, but not by 3‐HB (Figure 6b). Therefore, we suggested that EcNP3 plays its role in regulating IL‐10 production through GPR43.

### 3.8 The regulatory effect of EcNP3 on IL‐10 via histone deacetylases

Propionate, a member of SCFAs, is a common histone deacetylase (HDAC) inhibitor. Propionate typically acts by inhibiting class I HDACs,^40^ which include HDAC1, HDAC2, HDAC3, and HDAC8. The regulation of acetylation is related to gene expression. Acetylation promotes chromatin unhelix, thereby promoting gene transcription and translation. pan‐HDAC inhibitor Trichostatin A (20 nM), HDAC1 inhibitor CI994 (5 μM), HDAC2 inhibitor CAY‐10683 (100 nM), HDAC3 inhibitor RGFP996 (5 μM), and HDAC8 inhibitor PCI‐34051 (10 μM) were used to treat LPS‐induced RAW264.7 cells for 24 h, respectively. To explore whether the promotion effect of EcNP3 on IL‐10 production is related to the inhibition of HDAC and the regulation of acetylation level. The results showed that Trichostatin A and CI994 significantly upregulated the IL‐10 expression, while the other HDAC inhibitors had no significant effect (Figure 6c). Therefore, we further examined the expression and acetylation level of HDAC1 in RAW264.7 cells after EcNP3 treatment. The results showed that EcNP3 downregulated the expression of HDAC1 protein and promoted the acetylation level of histone 3(Figure 6d). Therefore, EcNP3 might promote IL‐10 expression by inhibiting HDAC1 to promote gene acetylation levels.

## 4 DISCUSSION

In this study, the effects of propionate on anti‐inflammatory macrophages and their function were analyzed, and a new strategy to restore propionate levels in the intestine through engineered probiotics was explored, thereby alleviating colitis. We demonstrated that the engineered live biotherapeutic product EcNP3 was more effective in alleviating the symptom of DSS‐induced colitis than the chassis strains. EcNP3 alleviates DSS‐induced colitis by promoting mucus secretion, restoring peritoneal anti‐inflammatory macrophage depletion, and regulating macrophage immune function. Moreover, EcNP3 significantly promoted the IL‐10 cytokine production of anti‐inflammatory through GPR43/HDAC1 pathway for further regulating the balance between pro‐inflammatory and anti‐inflammatory cytokines.

EcN is used as a chassis strain, a probiotic strain generally recognized as harmless to humans and has demonstrated efficacy in alleviating IBD.^41^ Previous studies have shown that propionate has anti‐inflammatory effects,^42^ and by engineering EcN to produce propionate and colonize in the gut, a more sustained effect can be achieved compared to propionate alone. Our results suggested that EcNP3 is more effective than EcN in mitigating colitis symptoms in DSS‐induced colitis mice. Consequently, we concluded that the ability to produce propionate is a crucial factor in EcNP3's ability to alleviate colitis. These results implied that engineered bacteria could enhance the therapeutic effect of the original strain in several ways.^43^

Patients with colitis often exhibit impaired immune function, and the reduced protective mechanisms lead to excessive and persistent inflammation.^2^ Anti‐inflammatory macrophages produce anti‐inflammatory cytokines during inflammation, which limit inflammatory responses and promote tissue repair to maintain tissue homeostasis.^4^ Our research has confirmed that both propionate and EcNP3 promoted the expansion of anti‐inflammatory macrophages. In addition, the production of IL‐10 is an essential characteristic of anti‐inflammatory macrophages. Cytokine analysis and real‐time quantitative PCR assays showed that compared with the EcN treatment group, the expression level of IL‐10 was significantly increased in the EcNP3 treatment group, while there was no significant difference in other cytokines. Therefore, we believe that EcNP3 can achieve immunomodulatory and improve intestinal mucosal integrity by promoting IL‐10 cytokine expression and cooperating with the prebiotic function of the chassis cell (*E. coli* Nissle 1917). It has been shown that IL‐10 could promote correct protein folding in goblet cells under adverse conditions, maintaining mucin production and secretion by inhibiting ER stress and UPR activation.^44^ Moreover, our study also demonstrated that EcNP3 could improve the loss of goblet cells and the production of MUC‐2 in DSS‐induced colitis mice. However, whether the protection of EcNP3 on mucus barrier depended on its promotion of IL‐10 needs to be further explored.

HDAC is a crucial receptor for propionate. In other intestinal tract‐related studies, propionate promoted the migration, renewal, and repair of intestinal epithelial cells by inhibiting class I histone deacetylases.^40^ Propionate also upregulated the transcription of heat shock protein 70 (HSP70) in intestinal Caco‐2 cells through HDAC inhibition, which increased the integrity and viability of colonic epithelial cells and maintained intestinal homeostasis.^45^ However, limited studies on regulating intestinal immunity by propionate via HDAC inhibition exist. In this study, we aimed to investigate the immunomodulatory role of EcNP3 by inhibiting HDAC activity by propionate. As expected, EcNP3 upregulates the expression of IL‐10 in anti‐inflammatory macrophages by inhibiting HDCA1, thereby affecting intestinal homeostasis. However, the more detailed epigenetic mechanism between HDAC1 inhibition and IL‐10 upregulation in macrophages needs to be further investigated.

## 5 CONCLUSION

This study provided a theoretical framework for the potential use of propionate‐producing probiotic EcNP3 as a novel treatment strategy for IBD treatment. Engineered live biotherapeutic products can enhance the therapeutic effect of probiotics by heterologous expression of nutrients or drugs while maintaining the probiotic function of chassis strain in regulating intestinal flora.^31^, ^46^ The development of eLBPs presents novel avenues for future disease treatment strategies.^47^ Further investigations are warranted to determine the feasibility and clinical applicability of EcNP3 in the management of IBD.

## AUTHOR CONTRIBUTIONS

**Guangbo Kang:** Data curation; formal analysis; funding acquisition; methodology; project administration; validation; writing – original draft; writing – review and editing. **Xiaoli Wang:** Conceptualization; data curation; formal analysis; methodology; validation; visualization; writing – original draft; writing – review and editing. **Mengxue Gao:** Conceptualization; data curation; formal analysis; methodology; validation; writing – original draft. **Lina Wang:** Investigation; methodology; validation; visualization. **Zelin Feng:** Investigation; validation; visualization. **Shuxian Meng:** Investigation; validation; visualization. **Jiahao Wu:** Investigation; validation; visualization. **Zhixin Zhu:** Investigation; validation; visualization. **Xinran Gao:** Investigation; validation; visualization. **Xiaocang Cao:** Conceptualization; formal analysis; funding acquisition; investigation; resources; supervision; writing – original draft; writing – review and editing. **He Huang:** Conceptualization; formal analysis; funding acquisition; investigation; project administration; resources; supervision; writing – review and editing.

## CONFLICT OF INTEREST STATEMENT

The authors have declared that no competing interest exists.

## Supporting information


**Table S1.** Strains and plasmids used in this study.
**Table S2.** Primers used for gene editing and N_20_ sequences.
**Table S3.** Disease Activity Index (DAI) scoring system.
**Table S4.** Primers used for gene expression levels in this study.

## Data Availability

Data will be made available on request.
