# Supplementary material for: Propionate‐producing engineered probiotics ameliorated murine ulcerative colitis by restoring anti‐inflammatory macrophage via the GPR43/HDAC1/IL‐10 axis
Source: Bioeng Transl Med. 2024 May 27;9(5):e10682. doi: 10.1002/btm2.10682 (PMC11561831; doi:10.1002/btm2.10682)
Supplement: Supplementary file 1 — Table S1. Strains and plasmids used in this study. Table S2. Primers used for gene editing and N20 sequences. Table S3. Disease Activity Index (DAI) scoring system. Table S4. Primers used for gene expression levels in this study. [file BTM2-9-e10682-s001.docx]

Table S1. Strains and plasmids used in this study

| Designation | Genotype | Reference |
| --- | --- | --- |
| Strains |  |  |
| *E. coli* DH5α | F-φ80 lac ZΔM15 Δ(lacZYA-arg F) U169 endA1 recA1 | TransGen Biotech |
| *E. coli* Nissle 1917 |  | biobw |
| EcNP1 | pCDFDuet-*IdhA*-*pct*；pETDuet-*lcdA-acul* | This study |
| EcNP2 | pCDFDuet-*IdhA*-*pct*；pETDuet-*lcdA-acul*, *ΔpflB* | This study |
| EcNP3 | pCDFDuet-*IdhA*-*pct*；pETDuet-*lcdA-acul;* *ΔpflB; ΔpoxB* | This study |
| EcNP4 | pETDuet-*IdhA*-*pct*；pCDFDuet-*lcdA-acul;* *ΔpflB; ΔpoxB; ΔackA* | This study |
| Plasmids |  |  |
| pETDuet | pBR322-derived ColE1 replicon; lac-inducible expression; amp | our laboratory |
| pCDFDuet | CloDF13 replicon; lac-inducible expression; streptomycin | our laboratory |
| pETDuet-*IdhA*-*pct* | pBR322-derived ColE1 replicon; lac-inducible expression; *Idh; pct*; amp | This study |
| pCDFDuet-*lcdA-acul* | CloDF13 replicon; lac-inducible expression; *lcdA; acul* ; streptomycin | This study |
| pCas | repA101(Ts); cas9; araB-inducible expression; Gam; Exo; Beta; lac-inducible expression; sgRNA-pMB1; kan | 62225* |
| pTargetF | pBR322-derived ColE1 replicon(pMB1) aadA; Spectinomycin | 62226* |
| pTargetF-*pflB* | pBR322-derived ColE1 replicon(pMB1) aadA; Spectinomycin; sgRNA-*pflB* | This study |
| pTargetF-*poxB* | pBR322-derived ColE1 replicon(pMB1) aadA; Spectinomycin; sgRNA-*poxB* | This study |
| pTargetF-*ackA* | pBR322-derived ColE1 replicon(pMB1) aadA; Spectinomycin; sgRNA-*ackA* | This study |

* Li Q, Sun B, Chen J, et al. A modified pCas/pTargetF system for CRISPR-Cas9-assisted genome editing in Escherichia coli. Acta Biochim Biophys Sin (Shanghai). 2021 Apr 15;53(5):620-627. doi: 10.1093/abbs/gmab036.

Table S2. Primers used for gene editing and N_20_ sequences

| Primer name/N20 | Sequence |
| --- | --- |
| pTargetF-L | gacttctacagcgcggagaatctcgctctc |
| pTargetF-R | ttctccgcgctgtagaagtcaccattgttg |
| L-sgRNA-*pflB* | GGAAGGTGGAGGTACGACCGcactagtattatacctaggac |
| S-sgRNA-*pflB* | CGGTCGTACCTCCACCTTCCgttttagagctagaaatagc |
| L-sgRNA-*poxB* | CGGTTCGATGGCTAACGCCAcactagtattatacctaggac |
| S-sgRNA-*poxB* | TGGCGTTAGCCATCGAACCGgttttagagctagaaatag |
| L-sgRNA-*ackA* | ATAGCAGTCAGCTGCGCAGAcactagtattatacctaggac |
| S-sgRNA-*ackA* | TCTGCGCAGCTGACTGCTATgttttagagctagaaatag |
| N_20_ |  |
| sgRNA-*pflB* | CGGTCGTACCTCCACCTTCC |
| sgRNA-*poxB* | TGGCGTTAGCCATCGAACCG |
| sgRNA-*ackA* | TCTGCGCAGCTGACTGCTAT |

Table S3. Disease Activity Index (DAI) scoring system

| Score | Weight loss (%) | Fecal consistency | Occult/Gross bleeding |
| --- | --- | --- | --- |
| 0 | 0 | Normal | Normal |
| 1 | 1-5 | Loose | Hemoccult positive |
| 2 | 5-10 |  |  |
| 3 | 10-20 |  |  |
| 4 | ＞20 | Diarrhoea | Gross bleeding |

 DAI = (Weight loss score + Fecal consistency score + Fecal bleeding score) / 3

Table S4 Primers used for gene expression levels in this study

| Gene | Prime sequences （5’→3’） |
| --- | --- |
| GAPDH | F：GGAGAAACCTGCCAAGTATG |
|  | R：TGGGAGTTGCTGTTGAAGTC |
| IL-10 | F：AGCCTTATCGGAAATGATCCAGT |
|  | R：GGCCTTGTAGACACCTTGGT |
| CD86 | F：CTGGACTCTACGACTTCACAATG |
|  | R：AGTTGGCGATCACTGACAGTT |
| iNOS | F：GCAAACATCACATTCAGATCCC |
|  | R：TCAGCCTCATGGTAAACACG |
| TNF-α | F：CTTCTGTCTACTGAACTTCGGG |
|  | R：CAGGCTTGTCACTCGAATTTTG |
| CD206 | F：GCTTCCGTCACCCTGTATGC |
|  | R：TCATCCGTGGTTCCATAGACC |
| Arg1 | F：AACACGGCAGTGGCTTTAAC |
|  | R：GTCAGTCCCTGGCTTATGGTT |
| CD163 | F：TGTGCAGTAACGGCTGGAG |
|  | R：ATCATGTTTGCAGTCCCAAAGA |
| CD301 | F：CAATGTGGTTAGTTGGATCGGC |
|  | R：CCCAGTTCTTAAAGCCTTTCTCA |
| IL-1RN | F：TAGACATGGTGCCTATTGACCT |
|  | R：TCGTGACTATAAGGGGCTCTTC |
| Fizz1 | F：CCCTGCTGGGATGACTGCTA |
|  | R：TGCAAGTATCTCCACTCTGGATCT |
| Ym1 | F：AGAAGGGAGTTTCAAACCTGGT |
|  | R：GTCTTGCTCATGTGTGTAAGTGA |
| IL-1β | F：GTGGCTGTGGAGAAGCTGTG |
|  | R：GAAGGTCCACGGGAAAGACAC |
| IL-6 | F：TGAGATCTACTCGGCAAACCTAGTG |
|  | R: CTTCGTAGAGAACAACATAAGTCAGATACC |
| CXCL1 | F：CTGGGATTCACCTCAAGAACATC |
|  | R：CAGGGTCAAGGCAAGCCTC |
| IL-2 | F：GTGCTCCTTGTCAACAGCG |
|  | R：GGGGAGTTTCAGGTTCCTGTA |
| IL-5 | F：TCAGGGGCTAGACATACTGAAG |
|  | R：CCAAGGAACTCTTGCAGGTAAT |
| GPR41 | F：CTTCTTTCTTGGCAATTACTGGC |
|  | R：CCGAAATGGTCAGGTTTAGCAA |
| GPR43 | F：ACCATCGTCATCATCGTTCA |
|  | R：ACGAAGCGCCAATAACAGAA |
